# Establishing a COVID‐19 pandemic severity assessment surveillance system in Ireland

**DOI:** 10.1111/irv.12890

**Published:** 2021-10-05

**Authors:** Lisa Domegan, Patricia Garvey, Maeve McEnery, Rachel Fiegenbaum, Elaine Brabazon, Keith Ian Quintyne, Lois O'Connor, John Cuddihy, Joan O'Donnell

**Affiliations:** ^1^ Health Service Executive Health Protection Surveillance Centre Dublin Ireland; ^2^ European Programme for Intervention Epidemiology Training (EPIET) European Centre for Disease Prevention and Control (ECDC) Stockholm Sweden; ^3^ Department of Public Health Health Service Executive North‐East Navan Ireland

**Keywords:** COVID‐19, pandemic, pandemic severity assessment, surveillance

## Abstract

We developed a COVID‐19 pandemic severity assessment (PSA) monitoring system in Ireland, in order to inform and improve public health preparedness, response and recovery. The system based on the World Health Organization (WHO) Pandemic Influenza Severity Assessment (PISA) project included a panel of surveillance parameters for the following indicators: transmissibility, impact and disease severity. Age‐specific thresholds were established for each parameter and data visualised using heat maps. The findings from the first pandemic wave in Ireland have shown that the WHO PISA system can be adapted for COVID‐19, providing a standardised tool for early warning and monitoring pandemic severity.

## 1 INTRODUCTION

Pandemic severity assessments (PSAs) provide information to determine the timing, scale and intensity of pandemics and to support decisions on the urgency of pandemic response actions and on implementing and lifting control measures.^1^, ^2^, ^3^ In the absence of a national surveillance system for assessing the severity of pandemics in Ireland, we adapted the WHO Pandemic Influenza Severity Assessment (PISA)^2^ methodology for COVID‐19. The WHO PISA project defined pandemic severity in terms of three indicators: transmissibility, seriousness of disease and impact. Transmissibility reflects the ease of transmission of viruses between individuals and communities. The seriousness of disease indicator describes the extent to which individual people get sick when infected. The impact indicator describes how the pandemic affects society (e.g., excess mortality) and the health‐care sector (e.g., hospitalisations).^2^ Following the declaration of the COVID‐19 pandemic on the 11 March 2020,^4^ we aimed to develop and implement a PSA monitoring system in Ireland, in order to inform public health preparedness, response and recovery measures, and to assist in improving the response to future waves of COVID‐19.

## 2 METHODS

A series of surveillance parameters for each indicator was identified and analysed by age group (0–14, 15–64 and ≥65 years), overall and week (Table 1). Threshold levels for each parameter were developed (by age group, overall and week) and applied to datasets from the first COVID‐19 wave (March–June 2020) in Ireland.

**TABLE 1:** Indicators and parameters used in the PSA monitoring system for Ireland, by type of data, date used for analysis/reporting and historic data used to establish thresholds

Indicators	Parameters	Type of data	Date used for analysis	Historic data used for thresholds
Transmissibility	Sentinel GP influenza‐like illness rate/100 000 population	Syndromic/clinical	GP phone consultation date	Sentinel GP ILI consultation data
	% GP OOHs flu calls (of total calls)	Syndromic	Date of call to GP OOHs service	GP OOHs flu calls data
	% GP OOHs cough calls (of total calls)			GP OOHs cough calls data
	COVID‐19 outbreaks (excluding family outbreaks)—number	Outbreak surveillance data	Date of outbreak notification	Influenza outbreak notifications
	COVID‐19 nursing home outbreaks—number	Outbreak surveillance data	Date of outbreak notification	
	SARS‐CoV‐2% positivity—NVRL‐UCD^a^	Laboratory testing data	Date of test	Influenza % positivity—NVRL‐UCD
	COVID‐19 confirmed cases—number	Notifications—lab confirmed only	Date of notification	Influenza confirmed case notifications
	COVID‐19 incidence per 100 000 population			
Impact	COVID‐19 hospitalised confirmed cases—number	Notifications—lab confirmed only	Date of notification	Confirmed influenza hospitalised notified cases
	COVID‐19 hospitalised confirmed cases—per 100 000 population			
	COVID‐19 confirmed ICU cases—number	Notifications—lab confirmed only	Date of notification	Confirmed influenza ICU notified cases
	COVID‐19 confirmed ICU cases—per 100 000 population			
	COVID‐19 confirmed cases that died—number	Notifications—lab confirmed only	Date of notification	Confirmed influenza case notifications that died
	COVID‐19 mortality rate per 1 000 000 population			
	Excess mortality—All cause (*Z* scores)	All cause death registrations	Date of death	All cause death registrations
Seriousness	COVID‐19 cumulative % cases hospitalised	Notifications—lab confirmed only	Cumulative by week of notification	% confirmed influenza cases hospitalised
	COVID‐19 cumulative % hospitalised cases admitted to ICU	Notifications—lab confirmed only	Cumulative by week of notification	% confirmed influenza hospitalised cases admitted to ICU
	COVID‐19 case fatality ratio (CFR)—all Cases	Notifications—lab confirmed only	Cumulative by week of notification	Influenza CFR—all cases (lab confirmed only)
	COVID‐19 CFR—hospitalised cases	Notifications—lab confirmed only	Cumulative by week of notification	Influenza CFR—hospitalised cases (lab confirmed only)
	COVID‐19 CFR—ICU cases	Notifications—lab confirmed only	Cumulative by week of notification	Influenza CFR—ICU cases (lab confirmed only)

^a^ The numerator is the total number of SARS‐CoV‐2 positive tests, and the denominator is the total number of tests for SARS‐CoV‐2 tested in the National Virus Reference Laboratory, University College Dublin (NVRL‐UCD).

Abbreviations: ICU, intensive care/critical care unit; ILI, influenza‐like illness; GP, general practice; OOH, out‐of‐hour; PSA, pandemic severity assessment.

Age‐specific statistical thresholds for transmissibility and impact parameters (baseline to extraordinary) were calculated using the Moving Epidemic Method based on historical data (previous 5 years).^5^ An upper ‘super‐extraordinary’ threshold level was established to compare peak pandemic activity of each pandemic wave. Threshold levels used to measure excess mortality (*Z* scores) were those used by the European Mortality Monitoring Project, EuroMOMO.^6^ Historical data from syndromic surveillance datasets such as general practice (GP) out‐of‐hours (OOHs) calls and sentinel GP influenza‐like illness (ILI) consultations were available for threshold calculation. In the absence of historical COVID‐19 data, historical influenza datasets were used as a proxy to calculate COVID‐19 thresholds. The thresholds for the seriousness of disease indicator were calculated based on means and standard deviations using cumulative historical influenza data for the previous five seasons on confirmed cases, hospitalisations and intensive care/critical care unit (ICU) admissions and deaths. Changes to national recommendations, case definitions, policy and testing strategies were documented to assist with data interpretation.

## 3 RESULTS

### 3.1 Transmissibility

**FIGURE 1:**
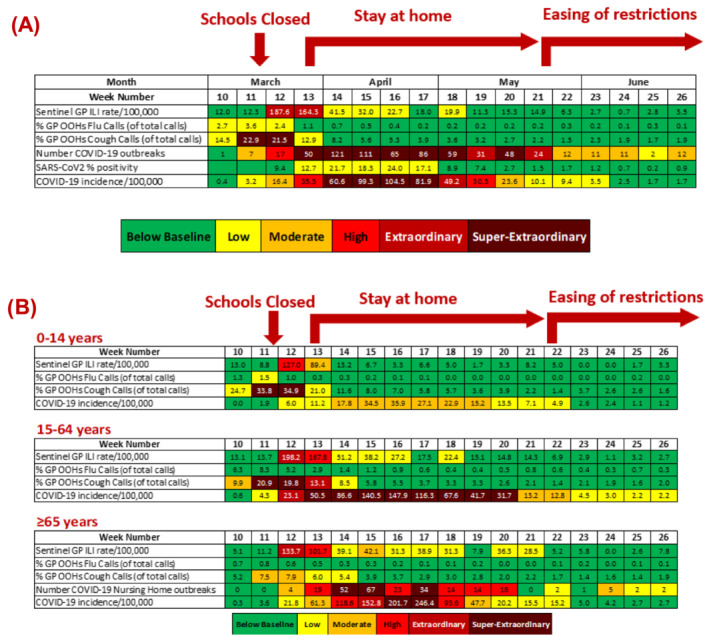
COVID‐19 transmissibility parameters by week and age group during the first COVID‐19 pandemic wave in Ireland, March–June 2020, colour coded by threshold level. (A) Overall and (B) by age group

During the first COVID‐19 pandemic wave (March–June 2020) in Ireland, GP OOHs cough calls first exceeded baseline levels during Week 10 2020 (week ending 08 March 2020) (Figure 1). COVID‐19 incidence per 100 000 population exceeded extraordinary levels for five consecutive weeks (30 March 2020 to 03 May 2020). The number of notified COVID‐19 outbreaks (excluding family outbreaks) exceeded baseline levels for 16 consecutive weeks and were at moderate levels by the end of June 2020, when restrictions were gradually eased over the 2020 summer.

Parameters used to monitor incidence in those aged less than 15 years old were below baseline levels or at low levels for most of the first COVID‐19 pandemic wave, with the exception of the proportion of GP OOHs cough calls which exceeded extraordinary levels for 2 weeks (09 March 2020 to 22 March 2020). The sentinel GP ILI consultation rate for the 0‐ to 14‐year age group increased considerably between Weeks 11 and 12 2020, followed by a decrease below baseline within 2 weeks of school closures (schools in Ireland closed on 12 March 2020 and remained closed for the academic year).

For the 15‐ to 64‐year age group, COVID‐19 incidence exceeded extraordinary levels for eight consecutive weeks, peaking at 175.7 per 100 000 population during Week 16 2020 (week ending 19 April 2020) and still remained above baseline levels at the end of the first COVID‐19 pandemic wave. COVID‐19 age specific incidence was highest for those aged 65 years and older, peaking at 242.3 per 100 000 population during Week 17 2020 (week ending 26 April 2020) and exceeding extraordinary levels for three consecutive weeks in April 2020.

### 3.2 Impact

**FIGURE 2:**
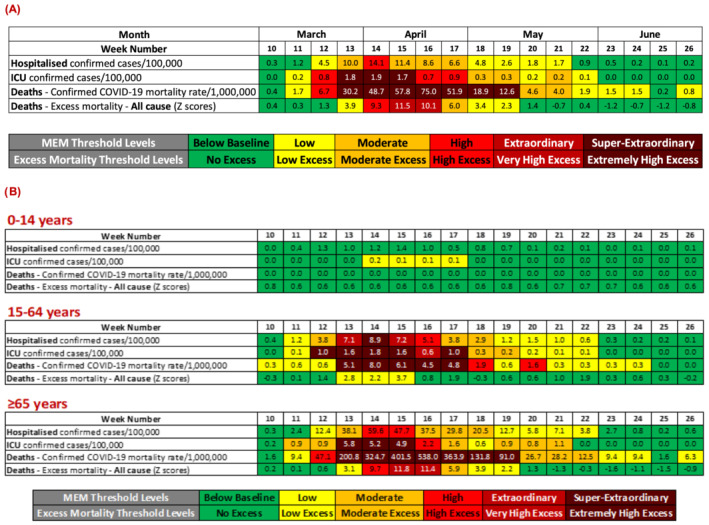
COVID‐19 impact parameters by week and age group, during the first COVID‐19 pandemic wave in Ireland, March–June 2020, colour coded by threshold level. (A) Overall and (B) by age group

The impact of the COVID‐19 pandemic during the first wave varied by age group. Levels were primarily below baseline for those aged 0–14 years and exceeded extraordinary levels for the 15‐ to 64‐year age group and for those aged 65 years and older (Figure 2). The impact on those aged 65 years and older was particularly severe, with mortality rates at very high levels for a period of nine consecutive weeks (exceeding extraordinary levels for seven consecutive weeks), peaking at 497 per 1 000 000 population during the week ending 19 April 2020. Excess all‐cause mortality was observed for seven consecutive weeks (late March to early May 2020) with the highest *Z* scores for excess deaths ever reported in Ireland. All impact parameters (hospitalisation and ICU admission rates) were below baseline for all ages by the end of the first pandemic wave, with the exception of the mortality rate which remained at low levels.

### 3.3 Seriousness of disease

By the end of the first COVID‐19 wave in Ireland, the cumulative proportion of hospitalised cases admitted to ICU was at extraordinary levels in those aged 15–64 years (19.0%) and for all ages (13.1%) and high for those aged 65 years and older (8.9%). Only five paediatric COVID‐19 cases were admitted to ICU during the first COVID‐19 pandemic wave.

The case fatality ratio (CFR) for all cases was at extraordinary levels for those aged 65 years and older (21.7%) and for all ages (6.0%) and at low levels for those aged 15–64 years (0.5%). There were no COVID‐19 deaths notified in the 0‐ to 14‐year age group in Ireland. The CFR in hospitalised cases was at extraordinary levels for those aged 65 years and older (34.7%) and for all ages (21.4%) and at high levels for those aged 15–64 years (5.9%).

## 4 DISCUSSION

A PSA surveillance system for COVID‐19 was established for Ireland. The panel of parameters selected enabled assessment of all indicators and age‐group differences for transmissibility, severity and impact and was based on an adapted WHO PISA model.^2^ Two parameters proved very timely and appeared sensitive for the early detection of COVID‐19, namely, data on GP OOHs cough calls and COVID‐19 ICU admission rates. GP syndromic parameters were sensitive and timely for the early detection of changing trends in paediatric age groups.

The PSA surveillance system is designed to be flexible. Parameters included may change over time as more suitable parameters are identified, such as sentinel GP SARS‐CoV‐2 positivity and CFR in nursing homes.^7^, ^8^ Homologous data for threshold calculation were used for some parameters (e.g., GP ILI consultations) and not for COVID‐19 specific parameters. The lack of historical COVID‐19 data for the development of thresholds is a limitation. As Ireland moves through successive COVID‐19 waves, one consideration is to use data from earlier waves to develop thresholds. WHO recommends inclusion of confidence levels when reporting PISA parameters,^2^ which would enhance data interpretation, in particular with limitations of available historical data for threshold calculation. There was a lower level of confidence in data reported during the initial weeks of the pandemic, due to the rapid evolution of the situation and frequent changes to testing capacity, criteria for testing and case definitions.

Our study provided an epidemiological description and assessment of the severity of the first COVID‐19 pandemic wave in Ireland. The heat maps were easily understood, concurred with the epidemiological situation and were reported to the National Public Health Emergency team. This PSA system will be used going forward in conjunction with enhanced surveillance data,^9^, ^10^ to monitor COVID‐19 activity in Ireland. We believe this is a useful surveillance tool to inform and guide national decisions and recommendations on public health interventions and for guiding control measures in Ireland as we move through pandemic waves. We have shown that the WHO PISA system can be adapted for COVID‐19 in Ireland (and possibly other pathogens with pandemic potential), providing a standardised tool to monitor pandemic severity and for early warning for current and future pandemic waves. Syndromic surveillance data are effective and timely when assessing pandemic severity, particularly when testing capacity may change and for monitoring novel respiratory pathogens (with no existing microbiological tests).

We recommend that PSAs, using this PSA system, be conducted regularly in Ireland as the pandemic progresses. We also recommend that other transmissibility measures such as reproductive numbers are considered for integration into the WHO PISA framework in the future. Current and future applications of this PSA system in Ireland include monitoring the impact of the COVID‐19 vaccination programme,^11^, ^12^ the changing epidemiology due to SARS‐CoV‐2 variants of concern^13^ and monitoring both SARS‐CoV‐2 and influenza each winter.

## AUTHOR CONTRIBUTIONS

**Lisa Domegan:** Investigation; conceptualization; methodology; formal analysis; writing‐review and editing. **Patricia Garvey:** Supervision; writing‐review and editing. **Maeve McEnery:** Data curation; validation; writing‐review and editing. **Rachel Fiegenbaum:** Data curation; validation; writing‐review and editing. **Elaine Brabazon:** Data curation; writing‐review and editing. **Keith Ian Quintyne:** Data curation; writing‐review and editing. **Lois O'Connor:** Supervision; writing‐review and editing. **John Cuddihy:** Supervision; writing‐review and editing. **Joan O'Donnell:** Supervision; writing‐review and editing.

## CONFLICT OF INTEREST

The authors have no conflicts of interest to declare that are relevant to the content of this article.

## ETHICS APPROVAL

Not required; aggregated anonymised routine surveillance data were used in this study.

## CONSENT TO PARTICIPATE

Not required; aggregated anonymised routine surveillance data were used in this study.

## CONSENT FOR PUBLICATION

Not applicable.

## CODE AVAILABILITY

Not applicable.

### PEER REVIEW

The peer review history for this article is available at https://publons.com/publon/10.1111/irv.12890.

## Data Availability

The data that support the findings of this study are available from the corresponding author, upon reasonable request.
